# Stereotactic body radiation therapy mitigates radiation induced lymphopenia in early stage non-small cell lung cancer

**DOI:** 10.1371/journal.pone.0241505

**Published:** 2020-11-30

**Authors:** Mark F. McLaughlin, Morshed Alam, Lynnette Smith, Jeffrey Ryckman, Chi Lin, Michael J. Baine

**Affiliations:** 1 Department of Radiation Oncology, University of Texas Southwestern, Dallas, Texas, United States of America; 2 Department of Statistics, University of Nebraska Medical Center, Omaha, Nebraska, United States of America; 3 University of Nebraska Medical Center, Department of Radiation Oncology, University of Nebraska Medical Center, Omaha, Nebraska, United States of America; West China hospital, Sichuan University, CHINA

## Abstract

**Introduction:**

Radiation-induced lymphopenia (RIL) occurs during treatment with conventional radiation in multiple organ sites. Development of RIL portends poor prognosis. Stereotactic body radiation therapy (SBRT) spares RIL in pancreatic cancer, but has not been examined in other sites commonly treated with SBRT. This work examines if SBRT similarly spares RIL in patients with non-small cell lung cancer (NSCLC).

**Materials and methods:**

Retrospective analysis was done at a single institution on 40 distinct cases of SBRT for early stage NSCLC from 2006–2017. Incidentally collected lymphocyte counts collected within 6 months of SBRT treatment were analyzed to determine if RIL occurred. The presence of RIL was correlated with location of initial failure and survival endpoints. Kaplan-Meier curves were constructed with significance defined at the level p < 0.05.

**Results:**

RIL was observed in 35% of the analyzed patients. Patterns of failure and survival data were comparable to prior SBRT literature. There was no observed association in two year local, nodal, or distant failure, progression free survival, or overall survival based on the presence of RIL.

**Discussion:**

SBRT spares RIL in NSCLC compared to historical rates observed with conventionally fractionated radiation. As understanding of the role of the immune system in cancer control continues to evolve, the importance of RIL sparing techniques take on increasing importance. This study represents further analysis of RIL sparing in SBRT in an early stage NSCLC cohort without the confounding influence of chemotherapy.

## Introduction

While radiation serves as an important pillar in oncology treatment, radiation therapy often comes with significant side effects. Patients treated with conventionally fractionated radiation for tumors located in the brain, spleen, lung, breast, pancreas, and head and neck show decreases in lymphocyte counts following therapy [1–8]. Importantly, this phenomenon, termed radiation-induced lymphopenia (RIL), directly correlates with decreased overall survival (OS) for patients with non-small cell lung cancer (NSCLC), pancreatic adenocarcinoma, and gliomas [1, 2, 9]. While it was initially theorized that RIL resulted from radiation exposure to sites of lymphocyte production or maturation, newer studies indicate that radiotoxic doses to the circulating lymphocytes themselves may be responsible [10, 11]. Post-radiation lymphopenia shows surprising persistence after the radiation event, taking up to two years for counts to normalize [5].

Modeling dose received by lymphocytes circulating throughout the body, traveling in and out of the radiation field, creates numerous difficulties. One model developed to measure radiation received during a standard 30 fraction glioma treatment estimates a mean dose of >2 Gy to circulating lymphocytes [12]. Experimental studies done extracorporeally indicate that a 2 Gy dose is sufficient to kill 60% of the lymphocyte population [13]. In mice, whole body exposures of as low as 0.3 Gy produce significant RIL while exposures of 0.5 Gy produce marked RIL [14]. Increasing the number of radiation fractions greatly increases the proportion of lymphocytes exposed to large doses of radiation as it increases the opportunities for the circulating blood pool to flow into the treatment field. Ergo, the model further suggests that hypofractionation may help mitigate decreases in lymphocyte count [12]. Research into stereotactic body radiation therapy (SBRT) for pancreatic cancer supports this hypothesis. While conventionally fractionated radiation induces treatment associated lymphopenia, SBRT therapies cause no significant decrease in lymphocyte counts [7, 15].

NSCLC represents an interesting study for RIL. As highly vascular organs, the lungs receive a large portion of blood flow at any given time. Prior studies demonstrate RIL with chemotherapy combined with conventionally fractionated (60 Gy in 30 fractions) radiation in stage III NSCLC. RIL occurs in 50% of the patients studied with an average reduction of 67%. RIL below 500 cells/mm^3^ corresponds with a non-significant decrease in median survival time from 27.3 to 21.8 months (p = 0.38) [2].

While historically treated with conventional fractionation, inoperable early stage NSCLC is increasingly treated definitively with SBRT, providing outcomes comparable to lobectomy [16]. Compared with conventional fractionation, SBRT delivers a higher dose per fraction in fewer fractions. Modern SBRT techniques compensate for respiratory motion, further reducing the volume of lung (and therefore volume of circulating blood pool) exposed to large doses of radiation. However, it is noteworthy that patients treated with SBRT may suffer from a large volume of low-dose spillage in healthy lung which receives a large proportion of the circulating blood pool. This spillage could theoretically expose a large fraction of blood to meaningful radiation doses.

This paper seeks to explore whether patients treated with SBRT for NSCLC experience RIL, here defined as two consecutive decreases in post-treatment lymphocyte count from baseline. Further analysis explores the significance of RIL on clinically relevant factors including patterns of recurrence and survival.

## Materials and methods

### Patient selection, treatment and data collection

Data was collected from 65 early stage NSCLC patients treated at a single institution between 2006 and 2017. Patient data was anonymized prior to access and analysis. The Institutional Review Board at the University of Nebraska Medical Center approved this study. Per policy of the local Institutional Review Board, informed consent was not required for analysis of anonymized patient data. SBRT radiation planning and treatment were performed under the supervision of board certified expert radiation oncologists. Patients were followed for a minimum of 1 year up to a maximum of 10 years. Baseline characteristics including age, sex, race, tumor histology, tumor size, performance status, and treatment dose/fractionation were recorded. Table 1 summarizes important demographic data of our patient population cohort. Treatment was delivered via megavoltage linear accelerators in four or five fractions with a median dose of 50 Gy. Incidentally collected lymphocyte counts were recorded for up to three instances within the six months preceding treatment and averaged to establish a baseline. Up to six post-treatment lymphocyte counts were collected within 6 months following treatment and used to monitor response. Patients were excluded from analysis for concomitant factors associated with alteration of lymphocyte count such as chemotherapy, steroid use, or unrelated infection. Further, patients without a baseline lymphocyte measurement or with one or fewer post-treatment lymphocyte counts were excluded. The location of failure was recorded for patterns of failure analysis. In patients with multiple lesions treated, only the initial treatment was analyzed with subsequent treatments taken as evidence of recurrence.

**Table 1:** Demographic and treatment characteristics.

Characteristics	Specifics	Total	RIL Change		P-value
			Decreasing 14(35%)	Non-decreasing 26(65%)	
Age in Years (median, IQR)	-	69.17 (59–76)	71.33 (62–76)	67.55 (59–76)	0.263
SBRT dose Gy (median, IQR)	-	50.0 (48–50)	49.0 (48–50)	50.0 (48–50)	0.250
Gender	Male	45%	12.5%	32.5%	0.510
	Female	55%	22.5%	32.5%	
Race	White	85%	27.5%	57.5%	0.651
	Black	10%	5.0%	5.0%	
	Others	5%	2.5%	2.5%	
Histology	Adeno	55%	20.0%	35.0%	0.232
	Squamous	17.5%	10.0%	7.5%	
	Others	27.5%	5.0%	22.5%	
Tumor size	< = 2 cm	53.85%	12.82%	41.03%	0.108
	>2 cm	46.15%	23.08%	23.08%	
Eastern Cooperative Oncology Group (ECOG) status	(0–1)	80%	30.0%	50.0%	0.689
	(2–3)	20%	5.0%	15.0%	
Number of fractions	4	32.5%	15.0%	17.5%	0.480
	5	67.50%	20.0%	47.5%	

IQR = Interquartile range.

### Statistical analysis

The initial SBRT treatment was selected for analysis for each patient. RIL was defined as a reduction in lymphocyte count in two consecutive blood draws within the first 6 months following treatment. Progression free survival (PFS) and OS time was calculated from the date of diagnosis to death. Time to local failure (LF), nodal failure (NF), and distant metastasis (DM) was calculated from the date of initial treatment to the date of the respective events.

As a part of our descriptive analysis mean, median, and interquartile range were calculated for continuous variables. Frequencies and percentages were calculated for categorical variables. Wilcoxon rank-sum test was used to assess the relationship between RIL and continuous variables while chi square and Fisher’s exact test were used to assess the relationship with categorical variables. Kaplan-Meier curves were constructed for LF, NF, DM, PFS, and OS with respect to RIL. Individual lymphocyte trend plots were constructed by using statistical software package R with all other statistical analysis using SAS software. P-values less than 0.05 for any test was considered as statistically significant. Patient data is available in the S1–S3 Tables.

## Results

### Demographics

Of the 65 patients analyzed, 25 were excluded for a final cohort consisting of 40 patients. Demographics of the cohort reflected the neighboring community of the single institution performing the study. The vast majority of patients were white. A small majority was female. Adenocarcinoma was the most common histologic subtype, followed by squamous cell, then all other subtypes. Treatment was most commonly done in five fractions (67.5%) though some patients received four. A slight majority of patients had T1 disease (tumor maximum diameter ≤ 3 cm) as opposed to T2 (maximum diameter > 3 cm but ≤ 5 cm) or higher. The majority of patients had good performance status (80% ECOG 0–1). There was no significant association between decreases in lymphocyte count and any demographic characteristics such as patient age, gender, race, histology, tumor size, ECOG status, or number of treatment fractions. In our cohort, 14 patients (35%) had evidence of RIL following treatment while 26 patients (65%) showed no evidence of RIL. Median average baseline total lymphocytes measured 1,200 cells/mm^3^ (range 400–4,700 cells/mm^3^) while median average post-treatment lymphocytes measured 850 cells/mm^3^ (range 240–2,200 cells/mm^3^).

### Effect of RIL on tumor progression

**Fig 1:**
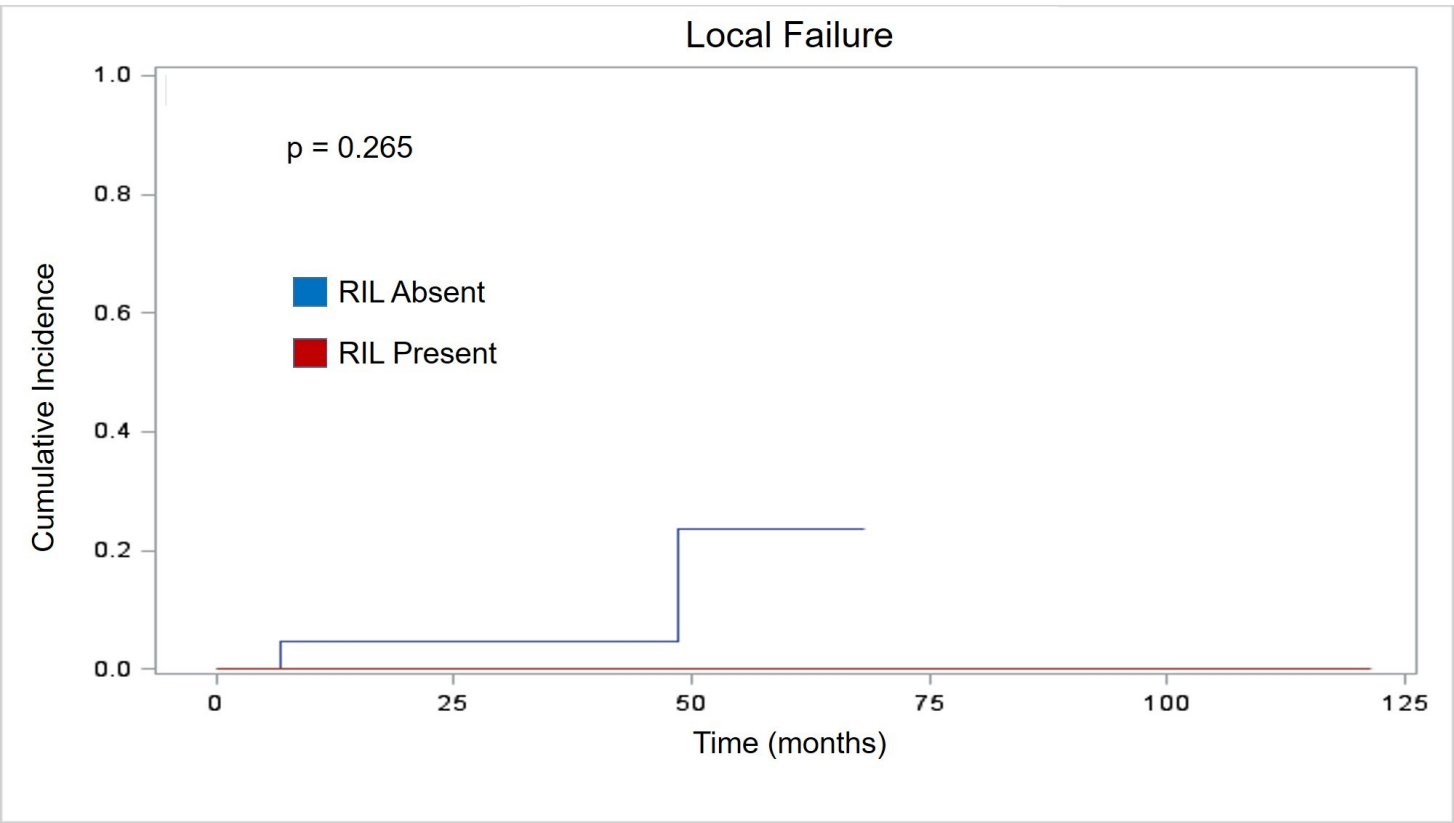
Local failure. Cumulative incidence curve showing no statistical difference in time to local failure (months) based on RIL status.

**Fig 2:**
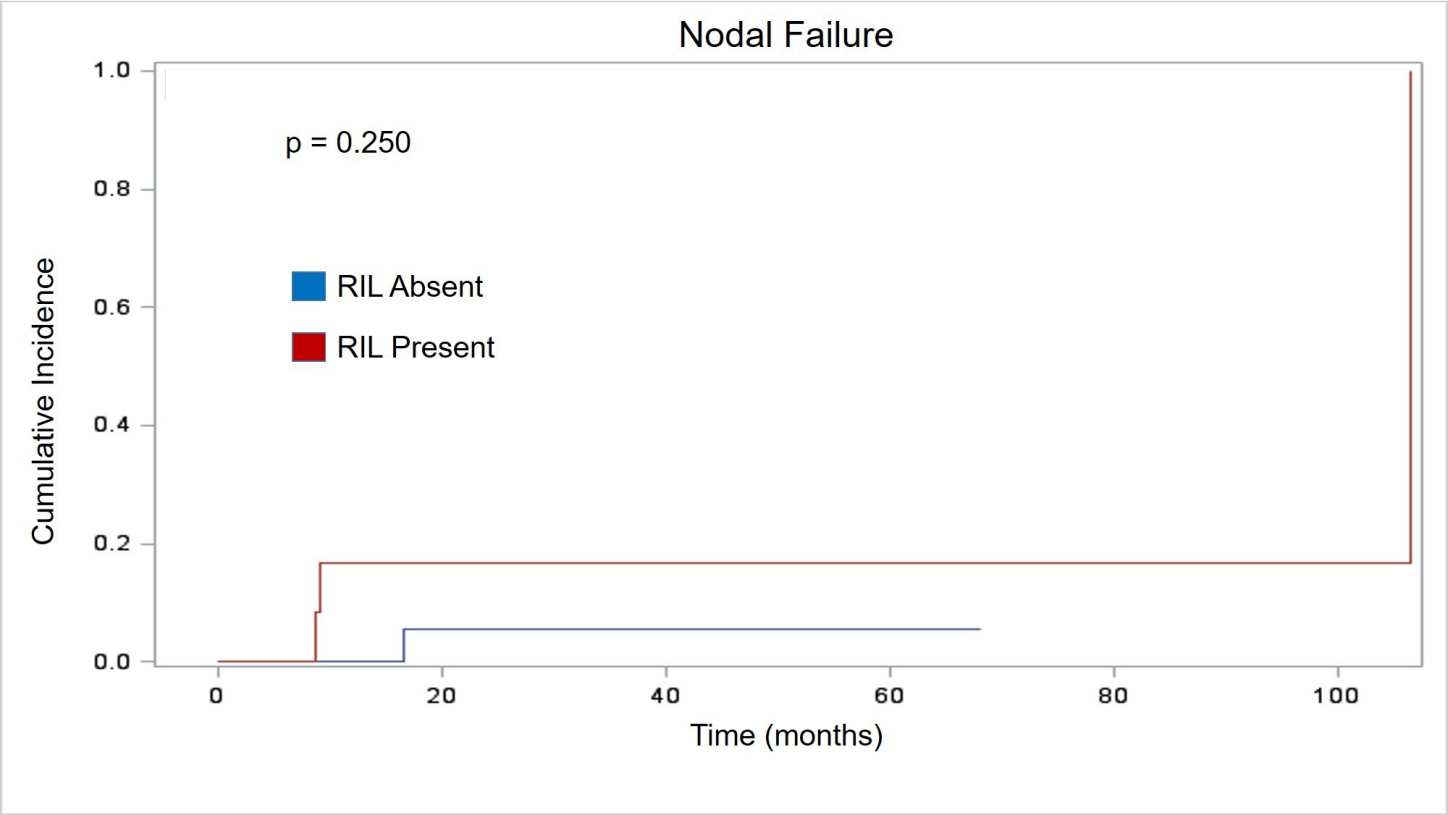
Nodal failure. Cumulative incidence curve showing no statistical difference in time to nodal failure (months) based on RIL status.

**Fig 3:**
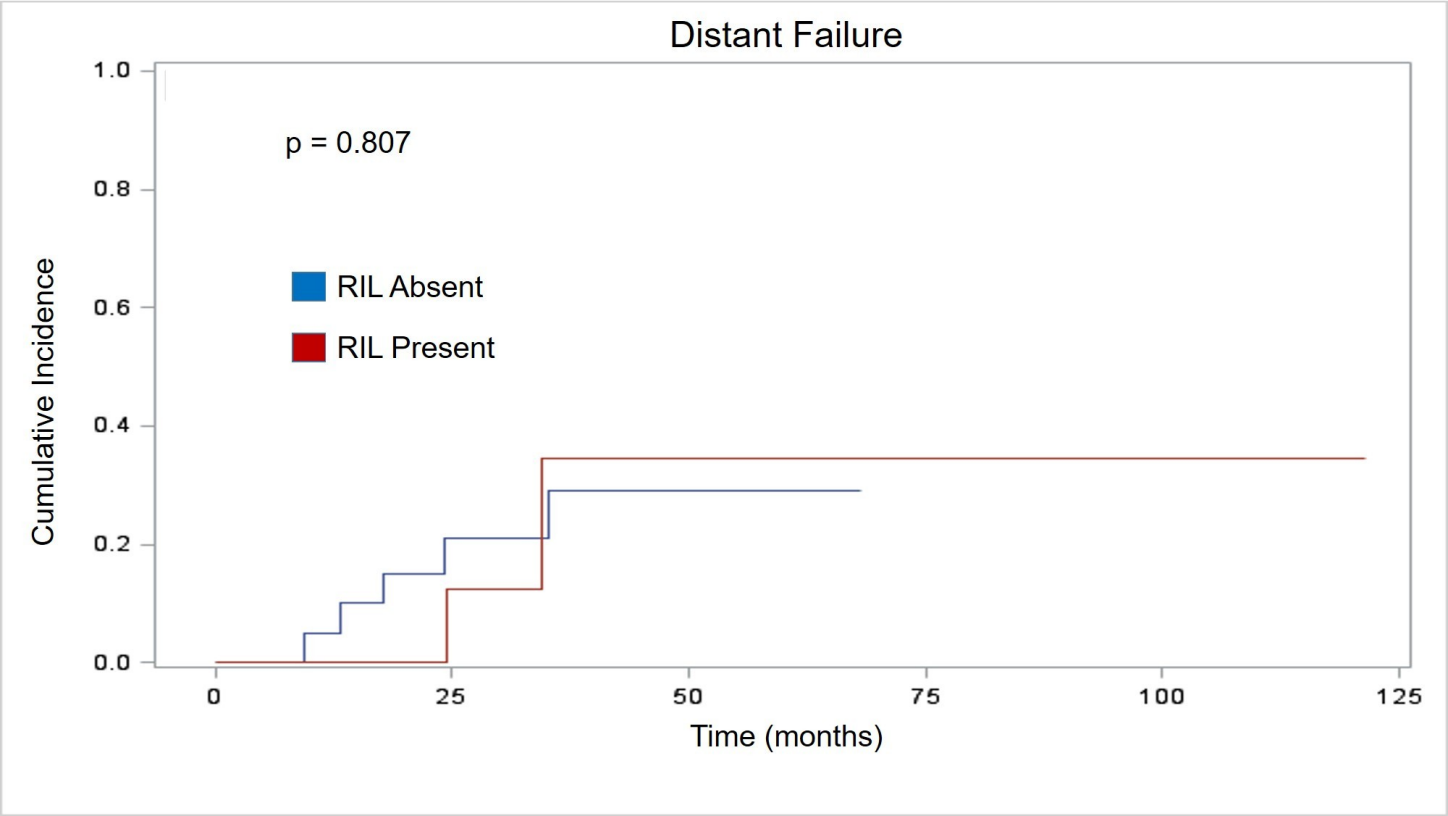
Distant failure. Cumulative incidence curve showing no statistical difference in time to distant failure (months) based on RIL status.

The presence of RIL had no statistically significant effect on patterns of failure. Similar to established literature for NSCLC treated with SBRT, local failure rates were rare in our cohort with 95% local control at two years. Two patients developed isolated local failure as the first site of relapse. Patients with stable/increasing lymphocyte counts had no significant difference in two-year local failure when compared with patients with decreasing lymphocyte counts (p = 0.265). Fig 1 depicts graphical representation of time to LF. While NF and DM were more common than LF, they remained a small percentage of the overall cohort. Four patients developed NF as their first site of relapse. Two-year NF also showed no significant association with RIL (p = 0.25). Detailed timing of NF is illustrated in Fig 2. Similarly, analysis of DM showed no significant association with RIL (p = 0.807) in the six patients where DM was the first site of failure. Specific analysis of distant failure is seen in Fig 3.

### Effect of RIL on survival

**Fig 4:**
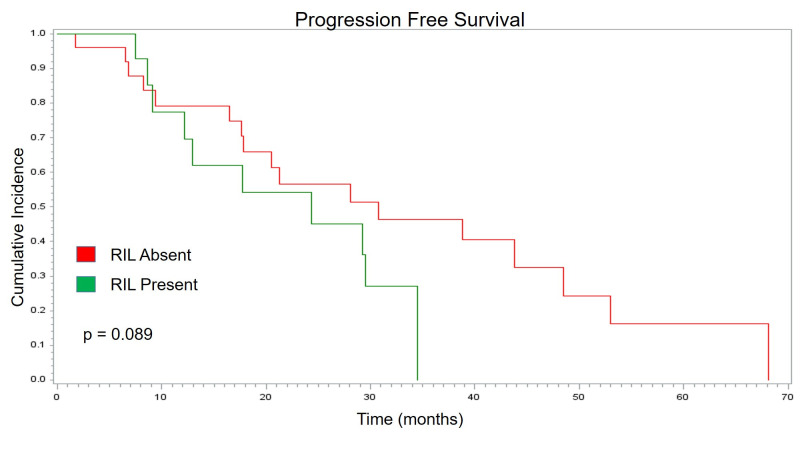
Progression free survival. Kaplan Meier curve showing no statistical difference in progression free survival (months) based on RIL status.

**Fig 5:**
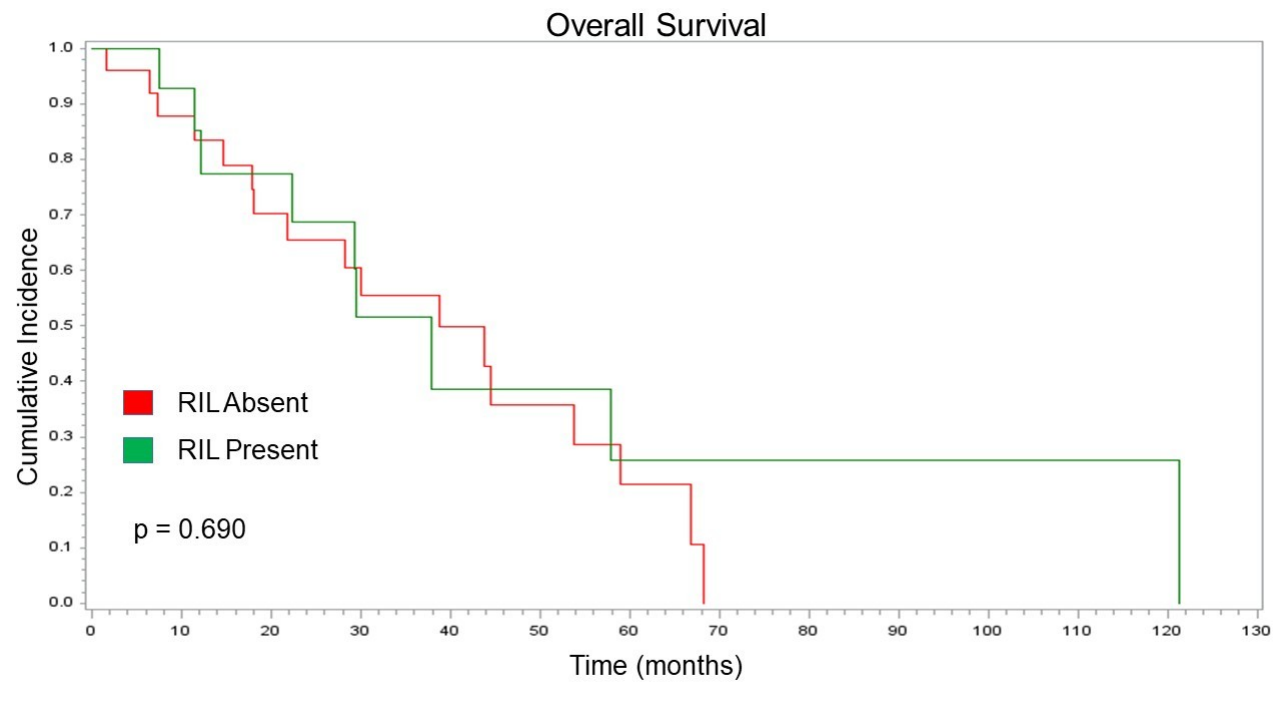
Overall survival. Kaplan Meier curve showing no statistical difference in overall survival (months) based on RIL status.

As each component of progression showed no change based on RIL status, PFS also showed no significant association with RIL (p = 0.089). Detailed PFS trends based on the presence of RIL are illustrated via Kaplan-Meier curve in Fig 4. Patients with stable/increasing lymphocyte counts had no significant difference OS when compared with patients with decreasing lymphocyte counts (p = 0.690). Two-year PFS and OS was nominally longer in those whose lymphocyte count did not decrease, though again this difference was non-significant. This non-significant difference is illustrated in Fig 5. Table 2 summarizes the patterns of failure and survival analysis in this study.

**Table 2:** Effect of RIL on 2-year cumulative incidence and 2-year survival.

	RIL Present 2-year estimate (95% CI)	RIL Absent 2-year estimate (95% CI)	P-value
Cumulative incidence of local failure	0% (NE)	4.5% (0.0–19.4%)	0.265
Cumulative incidence of nodal failure	16.7% (2.4–42.5%)	5.6% (0.0–23.6%)	0.250
Cumulative incidence of distant failure	0% (NE)^a^	15.0% (3.6–34.0%)	0.807
Progression-Free Survival	54.2% (25.0–76.2%)	56.6% (34.2–73.9%)	0.089
Overall Survival	65.6% (42.6–81.0%)	68.7% (36.4–87.1%)	0.690

^a^NE = Non-estimable.

## Discussion

This work represents further investigation of RIL following SBRT for NSCLC. Our results suggest that SBRT may not induce clinically meaningful RIL despite the associated risk of a large volume of healthy lung receiving low-dose spillage. Our results differ from those recently published by Zhao et al., where RIL was associated with both lower PFS and OS in patients treated with SBRT for early stage NSCLC [17]. One major explanatory difference lies in the timing of lymphocyte collection, with Zhao’s analysis focusing on lymphocyte counts collected within a week of SBRT completion and this analysis focusing on a period of up to 6 months post-treatment. Further, the majority of patients in Zhao’s analysis received a 10 fraction treatment, compared to the 4–5 fractions used in this study. Both our study and Zhao’s analysis utilize radiation alone, as opposed to previous analysis in advanced stage lung cancer which utilized chemotherapy followed by radiation [2]. An additional recent study by Chen et al. comparing immunotherapy combined with SBRT (50 Gy in 4 fractions or 60 Gy in 10 fractions) or traditional fractionation (45 Gy in 15 fractions) found that only the traditional fractionation scheme was associated with absolute lymphocyte decline [18].

These results are in line with predicted improvements in lymphopenia with hypofractionated regimens [12] and previously reported studies with SBRT in pancreatic cancer [15]. In contrast to conventionally fractionated radiation which decreased lymphocyte counts by 67% from baseline with 49% of patient developing severe lymphopenia (<500 cells/mm^3^) [2], our analysis found a median decline of only 29% with 22.5% developing severe lymphopenia. This comparison comes with the caveat that the conventionally fractionated patients additionally received chemotherapy and had stage III lung cancer.

Lymphopenia can impact progression and survival in multiple ways. Immunocompromised patients carry elevated risks for infection. In our cohort only one patient had infection as cause of death. This low infectious risk aligns with our finding that a minority of patients had decreased lymphocyte counts and even fewer had severe lymphopenia. However, previous studies using conventionally fractionated radiation which did show significant RIL also had low numbers of deaths due to infection [2]. Differences in clinical outcomes must therefore be driven by factors other than opportunistic infections. It is becoming increasingly clear that the immune system plays a role in preventing metastasis and growth of tumors [19]. Despite this growing body of evidence, no significant difference was found in two-year local, nodal, or distant failure between groups with stable/increasing lymphocyte counts and those with decreasing lymphocyte counts in our cohort.

Numerous novel and emerging cancer treatments rely on the immune system to recognize and eliminate cancer cells. In NSCLC, the potential exists to combine immunotherapy with existing radiation treatments. If radiation were to cause significant RIL, it could blunt the immune system’s ability to respond to tumor cells. This work suggests that RIL would not be a significant confounding factor for NSCLC patients treated with SBRT combined with immunotherapy.

Importantly, unlike many previous studies which combine some form of chemotherapy with radiation, this work deals with patients predominantly receiving definitive radiotherapy only, as is the standard paradigm. This removes many confounding effects as many chemotherapeutic regimens prove quite cytotoxic to circulating lymphocytes.

While this study supports the continued use of SBRT in lung cancer without modification for lymphopenia precautions, there are some limits to its scope and applicability. As a retrospective study, it does not account for potential confounding variables. Also, lymphocyte counts were not taken at uniform times pre- and post-irradiation. Time-dependent and self-resolving phenomenon could therefore have been missed in our analysis. Demographics of the single institution patient population used in the study reflect the relatively ethnically homogenous population of the area served. This may limit applicability of results to broader sections of the population. It is unknown if members of non-white ethnic groups might respond differently to radiation. Further, as a single institution study, overall numbers are relatively low. Conversely, the single institution nature lends itself to reproducibility with regards to patient setup, treatment, and ancillary care.

The results presented here support the notion that modern SBRT techniques, in contrast to their predecessors, do not significantly alter circulating lymphocyte counts. Further, patients who experienced decreasing lymphocyte counts after SBRT therapies exhibited no significant difference in clinical outcomes. Further work will outline the significance of dose parameters such as lung V5 to lymphopenia and recurrence patterns.

## Conclusions

Similar to studies of pancreatic cancer, SBRT spares clinically significant RIL from a majority of patients with early stage NSCLC.

## Supporting information

S1 Table Patient information. Patient-level data forming the basis of this analysis.
(XLSX)

S2 Table Patient information. Patient-level data forming the basis of this analysis.
(XLSX)

S3 Table Patient information. Patient-level data forming the basis of this analysis.
(XLSX)
